# The role of *Odontella aurita*, a marine diatom rich in EPA, as a dietary supplement in dyslipidemia, platelet function and oxidative stress in high-fat fed rats

**DOI:** 10.1186/1476-511X-11-147

**Published:** 2012-10-31

**Authors:** Adil Haimeur, Lionel Ulmann, Virginie Mimouni, Frédérique Guéno, Fabienne Pineau-Vincent, Nadia Meskini, Gérard Tremblin

**Affiliations:** 1 Université du Maine, PRES L’UNAM, EA 2160 MMS (Mer, Molécules, Santé), Faculté des Sciences et Techniques, Le Mans - IUT Département Génie Biologique, Laval, France; 2 Université Hassan II, Laboratoire de Biochimie, Environnement et Agroalimentaire, Faculté des Sciences et Techniques, Mohammedia, Maroc; 3 Centre Hospitalier du Mans, Laboratoire d’Hémostase, Le Mans, France

**Keywords:** *Odontella aurita*, *n*-3 PUFA, Platelet aggregation, Metabolic syndrome, Oxidative stress

## Abstract

**Background:**

Dietary changes are a major factor in determining cardiovascular risk. *n*-3 polyunsaturated fatty acids modulate the risk factors for metabolic syndrome via multiple mechanisms, including the regulation of the lipid metabolism. We therefore investigated the effect of *Odontella aurita*, a microalga rich in EPA, which is already used as a food supplement, on the risk factors for high-fat diet induced metabolic syndrome in rats.

**Methods:**

Male Wistar rats were divided into 4 groups and were fed with a standard diet (control); with the standard diet supplemented with 3% freeze-dried *O. aurita* (COA); with a high-fat diet (HF); or with the high-fat diet supplemented with 3% of freeze-dried *O. aurita* (HFOA) for 7 weeks. In this study we evaluated the impact of these different diets on the risk factors for metabolic syndrome, such as hyperlipidemia, platelet aggregation, thromboxane B_2_ production, and oxidative stress.

**Results:**

After 7 weeks of treatment, high fat feeding had increased final body weight, glycemia, triacylglycerol, and total cholesterol levels in plasma and liver compared to the control diet. Collagen-induced platelet aggregation and basal platelet thromboxane B_2_ were also higher in the high-fat fed rats than in those in the control group. In the liver, oxidative stress was greater in the HF group than in the control group. *O. aurita* intake in HFOA-fed rats resulted in lower glycemia and lipid levels in the plasma and liver relative than in the HF group. Thus, in the HFOA group, *n*-3 polyunsaturated fatty acid levels in the tissues studied (plasma, liver, and platelets) were higher than in the HF group. Platelet hyper-aggregability tended to decrease in HFOA-fed rats as basal platelet thromboxane B_2_ production decreased. Finally, *O. aurita* reduced oxidative stress in the liver, with lower malondialdehyde levels and increased glutathione peroxidase activity.

**Conclusions:**

*O. aurita* is a marine diatom rich in EPA as well as in other bioactive molecules, such as pigments. The synergistic effect of these microalgal compounds, displayed a beneficial effect in reducing the risk factors for high-fat induced metabolic syndrome: hyperlipidemia, platelet aggregation, and oxidative stress.

## Background

Metabolic syndrome (MS) has usually been defined on the basis of a combination of risk factors for cardiovascular diseases (CVDs), such as impaired glucose metabolism, insulin resistance, atherogenic dyslipidemia, hypertension, and obesity [1]. Lifestyle modifications, including abundant calorie-rich food and physical inactivity are recent phenomena in human evolution and may be responsible for the onset of one or several risk factors for MS. Several studies have shown that animals fed high fat and/or high sucrose diets are more likely to develop risk factors for MS, such as dyslipidemia and hyperglycemia [2,3], insulin resistance [4], hepatic steatosis [5,6], platelet aggregation [7] and oxidative stress [8,9]. While the prevalence of MS is increasing worldwide [10], studies have shown that recent dietary modifications such as low fat diets, high-fiber diets, Mediterranean diets and diets rich in flavonoids and phenolic acids can reduce MS risk factors [11,12]. Another way to reduce risk in patients with MS may be to increase the relative abundance of omega-3 (*n*-3) polyunsaturated fatty acids (PUFAs) in the diet [13].

The relationship between dietary *n*-3 PUFA and the risk of developing cardiovascular diseases is described in epidemiological, clinical, and animal studies [14-16]. Eicosapentaenoic acid (EPA; 20:5n-3) and docosahexaenoic acid (DHA; 22:6n-3) are commonly referred to as bioactive *n*-3 fatty acids. They are known to reduce key risk factors for coronary diseases, for instance by reducing plasma triacylglycerol, arterial and venous thrombosis, diabetes mellitus, hypertension, and inflammation [15]. EPA and DHA also play a key role in normalizing platelet hyper-aggregability. When added to the diet, EPA and DHA can alter the phospholipid membrane composition of the cells, and therefore impact on the synthesis and action of eicosanoids, and regulate transcription factor activity and abundance. Another aspect of the action of *n*-3 fatty acids is to act as an important mediator of gene expression working via the peroxisome proliferator-activated receptors (PPARs) that control the expression of the genes involved in the lipid and glucose metabolism and adipogenesis [17]. Ethyl-EPA markedly reduced the fatty droplets in the liver cells of mice fed a high-fat diet, also lowering plasma levels of total cholesterol and triacylglycerols [18]. Kajikawa et al. [5] showed that oral administration of highly purified EPA ethyl ester (EPA-E) improved hepatic fat accumulation in high fat/high sucrose diet-fed mice by suppressing the triglyceride (TG) synthesis enzymes regulated by sterol regulatory element binding protein-1 (SREBP-1) and decreased the accumulation of hepatic monounsaturated fatty acids (MUFAs) produced by stearoyl-CoA desaturase 1 (SCD1). Adan et al. [7] showed that EPA and DHA feeding reduces serum cholesterol and triacylcerol levels, and decreases platelet aggregation in hypercholesterolemic rats.

The richest dietary source of EPA and DHA is fish oils. However, fish do not synthesize these fatty acids *de novo*, as they lack the required key enzymatic activities [19]. Instead, fish accumulate EPA and DHA primarily by consuming plankton and algae from the marine food chain [20]. Unfortunately fish also accumulate pollutants, the oil extracted from them also has unpleasant odor, and the proportion of specific fatty acids in lipids from this source are difficult to control. These drawbacks mean that an alternative and renewable source must be found.

Marine microalgae are the primary producers of *n*-3 PUFA in the marine food chain. So far, only a few species of algae have been approved for use as food complements, the one most studied is *Chlorella sp*., a fresh water unicellular green algae. It contains essential amino-acids, minerals, fibers, and lipid soluble vitamins [21,22]. Numerous studies have revealed the biochemical activities of *Chlorella*, especially when administered in the context of underlying disorders, such as streptozotocin-induced diabetes in rats [23]. Thus, in rabbits fed a high-cholesterol diet for 10 weeks, *Chlorella vulgaris*, demonstrated antilipidemic and antiatherosclerotic actions [24]. Cherng et al. [2] also showed that *Chlorella pyrenoidosa* has the ability to prevent dyslipidemia in rats and hamsters models fed a high-fat diet containing 20% hydrogenated coconut oil (as the source of saturated fatty acids). In a human study, Lee et al. [25] showed that in male Korean smokers, *Chlorella vulgaris* supplementation conserved plasma antioxidant nutrient status and improved erythrocyte antioxidant enzyme activities. *Chlorella* intake can also lower cholesterol levels in patients with hypercholesterolemia [26]. These potential health benefits of *Chlorella* have been attributed to the effects of specific ingredients in this microalga such as minerals, dietary fiber, proteins, and *n*-3 PUFA (18:3n-3). *Odontella aurita* is a microalgal marine diatom that is known to contain high levels of EPA (26% of total fatty acid) and several bioactive compounds, such as pigments, fibers and phytosterols, which have beneficial effects on human health [27,28]. *O. aurita* is currently approved for use as a dietary supplement. The studies of Moreau et al. [27] focus on carotenoids extracted from this microalgae, and show that an antiproliferative effect was produced in cultures of bronchopulmonary and epithelial cells when extracts were added to the cell medium. However, no nutritional studies have been yet conducted to demonstrate the biological effect of *O. aurita* as a food supplement or to investigate the possible synergistic effect of various microalgal compounds.

Against this background, the aims of this study are to investigate the effects of freeze-dried *O. aurita* as a food supplement on risk factors for high-fat induced MS in rats fed with a high-fat diet, principally hyperlipidemia in the bloodstream and liver, platelet aggregation and oxidative stress, in order to prevent cardiovascular diseases.

## Materials and methods

### Animal experiments and diet

Male Wistar rats weighing (120 ± 10 g) were purchased from (Janvier, CERJ, Le Genest Saint Isle, France) and housed in pairs in cages with a 12h light/dark cycle and maintained at 20 ± 2°C. Rats were fed an R-112 standard diet (SAFE, Augy, France) containing (on a per weight basis; 5% fat, 23% protein, 58% carbohydrates, 6% cellulose, 7% mineral, and 1% vitamins). After a 1-week acclimation period, the rats were randomly assigned to 4 groups, with 6 rats in each. The control group (C), continued to receive the standard diet (R-112). The second group (COA) was fed the standard diet supplemented with 3% (w/w) freeze-dried *O. aurita* (Innovalg, Bouin, France). The freeze dried *O. aurita* content in *n*-3 PUFA and pigments was as following: *n*-3 PUFA (in % of total fatty acids), total = 32%, EPA = 26%; pigments, total = 9 mg/g of dry matter, chlorophyll *a* = 30%, fucoxanthin = 20% of total pigments (results provided by Innovalg, Bouin, France). The third (HF) group received a high fat diet containing (on a per weight basis: 25% fat, 23% proteins, 38% carbohydrates, 6% cellulose, 7% mineral, and 1% vitamins). The fourth group (HFOA) was fed with high fat diet supplemented with 3% (w/w) of freeze-dried *O. aurita*. The composition of the four diets is shown in Table 1, and the fatty acid composition of the different diets is presented in Table 2. The rats had free access to food and water throughout the study. The bodyweight gain of the rats was monitored at regular intervals, and their daily food intake was estimated. All the experiments involving animals had been approved by the institutional ethics committee (Direction des Services Vétérinaires de la Mayenne, France, N° B53500). Rats were fed the test diets for 7 weeks and were anaesthetized (with Diazepam/Ketamine 4v/3v), following an overnight fast on day 49, and exsanguinated via the abdominal aorta. The liver and adipose tissue were removed, rinsed with ice-cold saline solution (0.9% NaCl) and stored at (-70°C).

**Table 1:** Diet composition

**Composition (g/kg)**	**C**	**COA**	**HF**	**HFOA**
Casein	230	230	230	230
Corn starch	200	200	130	130
Glucose	380	380	250	250
Cellulose	60	60	60	60
Lard	30	30	230	230
Corn oil	10	10	10	10
Rape oil	10	10	10	10
Mineral 205B SAFE	70	70	70	70
Vitamin 200 SAFE	10	10	10	10
freeze-dried *O. aurita*	-	30	-	30

Analysis was provided by SAFE (Scientific Animal Food & Engineering, Augy, France).

The mineral mixture provides the following amounts in mg/kg of diet: CaHPO_4_, 17.2; KCl, 4000; NaCl, 4000; MgO, 420; MgSO_4_, 2000; Fe_2_O_3_, 120; FeSO_4_, 7 H_2_O, 200; trace elements, 400. Trace element mixture (mg/kg of diet): MnSO_4_, H_2_O, 98; CuSO_4_, 5 H_2_O, 20; ZnSO_4_, H_2_O, 80; CoSO_4_, 7 H_2_O, 0.1; KI, 0.3.

The vitamin mixture provides the following amounts per kg of diet: retinol, 39.600 IU; cholecalciferol, 5000 IU; thiamin, 40 mg; riboflavin, 30 mg; pantothenic acid, 140 mg; pyridoxine, 20 mg; inositol, 300 mg; cyanocobolamin, 0.1 mg; ascorbic acid, 1600 mg; choline, 2.720 mg; folic acid, 10 mg; p-aminobenzoic acid, 100 mg; biotin, 0.6 mg.

C, control; COA, control + *O. aurita*; HF, high fat; HFOA, high fat + *O. aurita*.

**Table 2:** Dietary fatty acid composition

**Fatty acids (% molar)**	**C**	**COA**	**HF**	**HFOA**
14:0	0.94 ± 0.12	1.00 ± 0.15	1.50 ± 0.14	1.67 ± 0.19
16:0	17.16 ± 1.70	17.72 ± 1.86	24.58 ± 1.03	25.49 ± 1.53
16:1n-7	1.39 ± 0.05	2.41 ± 0.14	0.23 ± 0.06	0.23 ± 0.04
18:0	9.19 ± 0.16	8.53 ± 0.23	11.53 ± 0.31	11.29 ± 0.30
18:1n-7+n-9	45.02 ± 2.54	42.31 ± 2.81	42.69 ± 1,96	42.06 ± 2.12
18:2n-6	20.14 ± 1.81	18.86 ± 1.54	12.33 ± 0.47	11.92 ± 0.51
18:4n-3	0.4 ± 0.02	0.37 ± 0.03	0.22 ± 0.01	0.21 ± 0.01
20:4n-6	-	-	0.16 ± 0.02	0.15 ± 0.02
20:5n-3	-	0.83 ± 0.16	-	0.76 ± 0.11
22:6n-3	-	tr	-	tr

Total lipids were extracted from diets according to Folch et al. method [31]. Fatty acid methyl ester (FAMEs) were prepared according to the method of Slover and Lanza [33] and analyzed by gas chromatography as described in material and methods section. Values are expressed as mean ± SD (n=3). C, control; COA, control + *O. aurita*; HF, high fat; HFOA, high fat + *O. aurita*. tr: traces.

### Platelet aggregation assay

#### Platelet preparation

Blood was recovered in polyethylene tube containing Acid-Citric-Dextrose anticoagulant (9:1 v/v) (citric acid 130 mM, trisodium citrate 170 mM, dextrose 4%). Washed platelets were prepared as described previously by Mekhfi et al. [29] with some modifications. Briefly, blood was centrifuged at 230 × g for 15 min to obtain platelet rich plasma (PRP). The supernatant (PRP) was then centrifuged at 120 × g for 8 min to remove any residual erythrocytes and leukocytes. The platelet rich plasma obtained was acidified to pH 6.5 with citric acid 0.15 M to inhibit spontaneous aggregation. PRP was then centrifuged at 900 × g for 15 min to obtain platelet pellets. The cells were finally suspended in Tyrode buffer (NaCl 137 mM, KCl 2.6 mM, MgCl_2_ 0.9 mM, glucose 5.5 mM, gelatin 0.25%, HEPES 5 mM, CaCl_2_ 1.3 mM pH 7.4). Platelet poor plasma (PPP) was recovered for lipid analysis.

#### Platelet aggregation

Platelet aggregation was measured turbidimetrically in washed platelet suspensions [30] using an APACT 4004, 4-channel platelet aggregometer (LABiTEC, Ahrensburg, Germany). Platelet suspension concentrations were estimated by a Coulter cell counter (Beckman Coulter, Villepinte, France), and the concentrations were adjusted to 5×10^8^ cells/mL using Tyrode buffer. Aggregation tests were performed at 37°C in cuvettes stirred at 1000 rpm. Washed platelets (225 μL) were stimulated with ADP 10 μM (Sigma-Aldrich, Saint-Quentin Fallavier, France) or with Collagen (Kordia, Lille, France) 5 μg/mL. The light transmission was recorded for 5 min after platelet stimulation. The platelet aggregation was quantified as the maximum change in light transmission through a washed platelet solution expressed as a percentage of the light transmission through the blank (Tyrode buffer).

### Phospholipid fatty acid composition analysis

Total lipids were extracted from the liver with chloroform/methanol (2:1 v/v) according to the method of Folch et al. [31]. Furthermore total lipids were extracted from the platelets and plasma using the Bligh and Dyer method [32]. Phospholipids (PLs) and neutral lipids (NLs) were separated from the total lipids by solid phase extraction using silica gel columns Sep-pak (Sep-Pak plus, silica cartridges, Waters, France). Fatty acid methyl esters (FAMEs) were prepared according to the method of Slover and Lanza [33], and analyzed with a FOCUS gas-chromatography instrument (Thermo Electron Corporation, Les Ulis, France) equipped with a capillary column CP Sil-88 25 m × 0.25 mm id (Varian, Les Ulis, France). Analyses were carried out from 150°C to 220°C. The esters were detected with a flame-ionization detector. Individual fatty acids were identified from authentic fatty acid methyl ester standards (Sigma-Aldrich, Saint-Quentin Fallavier, France), and expressed as a percentage of total fatty acids.

### Biochemical analysis

Glycemia was measured in rat tails using a glucometer (FreeStyle PAPILLON mini, Abbott, Rungis, France). However, plasma triacylglycerol and total cholesterol levels were determined by enzymatic methods, using commercial Kits (Biomérieux S.A, Marcy l’Etoile, France). Triacylglycerol and total cholesterol levels were also determined in the liver using total lipids extracted by enzymatic methods.

### Measurement of lipid peroxidation in platelets and liver

Lipid peroxidation was evaluated by measuring the level of malondialdehyde (MDA) by the Ohkawa method [34]. 0.5 g of liver was homogenized with 4.5 mL KCl solution (1.15%). To 0.5 mL washed platelet solution or 0.1 mL liver homogenate was added 0.1 mL 8.1% sodium dodecyl sulfate (SDS), 0.75 mL acetic acid 20% pH 3.5, 0.75 mL thiobarbituric acid (TBA) 0.8% and distilled water to make the volume up to 2.5 mL. The tubes were placed in a water bath at 95°C for 60 min, and then immediately cooled in ice. 0.5 mL of water and 2.5 mL of a solution of *n*-butanol and pyridine (15:1 v/v) were added to each tube, which was then shaken vigorously and then centrifuged at 1000 × g for 10 min. The organic phase corresponding to the upper layer was aspired and the color intensity was measured at 532 nm. The standard range was prepared using the1,3,3,3- tetramethoxypropane (Sigma-Aldrich, Saint-Quentin Fallavier, France) solution.

### Glutathione peroxidase assay

The platelet content was released by sonication. The lysate was centrifuged at 4000 × g for 10 min and the supernatant was used to determine the platelet glutathione peroxidase activity. The liver was homogenized in KCl solution (1.15%) using a Potter tissue homogenizer then centrifuged at 4000 × g for 10 min. Liver glutathione peroxidase activity was measured in the supernatant.

The glutathione peroxidase (GPx) activity was determined according to Paglia and Valentine method [35] as modified by Chaudiére and Gérard [36]. Briefly, to a final volume of 1.5 mL containing Tris-HCl/EDTA (50 mM/0.1 mM) pH 7.6, NADPH,H^+^ (0.14 mM), reduced glutathione (GSH) (2 mM) and 0.7 U/mL of GSSG-reductase, was added 100 μL of platelet lysate or 100 μL of liver homogenate. The mixture was incubated at 37°C for 3 min. The reaction was started by adding 50 μL tertiary butyl hydroperoxide (*t*-BH) (0.2 mM). The change in absorbance was recorded at 340 nm for 5 min at 5 s intervals. An appropriate control without samples was run simultaneously. GPx activity was expressed in nmoles of hydroperoxide reduced per min and per mg protein.

### Protein assay

Protein was determined by Bradford’s colorimetric method [37] using Biorad reagent, with bovine serum albumin (Sigma-Aldrich, Saint-Quentin Fallavier, France) as standard.

### Thromboxane B_2_ measurement

Rat washed platelets underwent three successive freeze/thaw cycles to release the cell content. The basal TXB_2_ level was measured using an enzyme immunoassay (EIA) Kit (Enzo-Life sciences, Exeter, UK) according to the manufacturer’s recommendations.

### Statistical analysis

All values were expressed as the mean ± SD. After analysis of variance, the mean values were compared using Fisher's least significant difference test (Statgraphics Plus 5.1, Manugistics Inc., Rockville, MD, USA).

## Results

### Effect of diet treatments on body and organ weights

As shown in Table 3, the final bodyweight of the rats on the high-fat (HF) diet was significantly higher than that of the control (C) diet rats. Feeding with either the control or high-fat diet supplemented with *O. aurita* (COA and HFOA respectively) tended to decrease the final body weight after 7 weeks of diet treatment compared to both C and HF feeding, but this effect failed to reach statistical significance. With regard to organ weights (Table 3), only the adipose tissue was affected by the dietary treatments. The data showed that the HF diet significantly increased adipose tissue weight (*p* < 0.016) compared to the C diet. Adipose tissue weight/final body weight ratio (AT/Bw) was also calculated to evaluate the development of adiposity. The AT/Bw ratio was significantly greater in HF-fed rats than in the controls. The increased adiposity was prevented by *O. aurita* intake in the HFOA group, where the adipose tissue weight was reduced, and the AT/Bw ratio lower than that in HF-fed rats. Daily food intake was significantly greater in the C and COA groups than in the HF and HFOA groups. However, the daily energy intake did not differ for the different dietary treatments.

**Table 3:** Animal characteristics after 7 weeks of treatment

**Diet groups**	**C**	**COA**	**HF**	**HFOA**	***p *value**
**Organs (g)**					
Body weight (Bw)	359.1 ± 18.7^bc^	339.1 ± 27.8^c^	389.1 ± 13.1^a^	378.7 ± 27.4^ab^	0.008
Liver weight (Lw)	11.1 ± 1.5	10 ± 0.7	10.6 ± 0.6	11 ± 0.9	N.S
Adipose tissue (AT)	4.5 ± 1.6^b^	4.8 ± 1.1^b^	6.9 ± 0.8^a^	4.3 ± 0.9^b^	0.016
AT/Bw (%)	1.2 ± 0.5^b^	1.4 ± 0.3^ab^	1.8 ± 0.3^a^	1.1 ± 0.2^b^	0.045
Daily food intake (g/day)	46.2 ± 12.8^a^	42.8 ± 9.2^a^	35.9 ± 8.7^b^	32.3 ± 8.8^b^	< 0.001
Energy intake (Kcal/day)	170.47 ± 47.23	161.3 ± 34.7	168.23 ± 40.8	154.07 ± 41.97	N.S

Results are expressed as mean ± SD (n=5). After a one-way ANOVA, Student–Newman–Keuls (SNK) multiple comparison test, results are arranged in increasing order from left to right: a > b > c (*p* < 0.05). C, control; COA, control + *O. aurita*; HF, high fat; HFOA, high fat + *O. aurita*. N.S: Not significant.

### Effect of diet treatments on plasma and liver lipid levels

The *in-vivo* hypolipidemic and hypoglycemic effects of *O. aurita* intake was evaluated (Table 4). The HF group registered a significant increase in plasma glucose, triacylglycerols (TG) and total cholesterol compared to the C group (*p* < 0.009, *p* = 0.001 and *p* = 0.035 respectively). The presence of *O. aurita* produced a hypoglycemic and hypolipidemic effect in the HFOA-fed rats compared to the HF group. In the liver, the data revealed that liver lipids, especially TG and total cholesterol, were significantly greater in the HF group than in the controls (*p* < 0.001). The liver TG and total cholesterol levels were significantly lower in the HFOA-fed rats than in the HF group (Table 4).

**Table 4:** Glycemia and plasma and liver lipids determinations after 7 weeks of treatment

**Diet groups**	**C**	**COA**	**HF**	**HFOA**	***p *value**
**Plasma (mmol/L)**					
Glucose	4.30 ± 0.49^b^	3.96 ± 0.27^b^	5.17 ± 0.74^a^	4.57 ± 0.51^ab^	0.009
Triacylglycerols	0.84 ± 0.07^b^	0.90 ± 0.06^b^	1.06 ± 0.04^a^	0.91 ± 0.09^b^	0.001
Total cholesterol	1.49 ± 0.22^b^	1.57 ± 0.22^b^	1.87 ± 0.17^a^	1.51 ± 0.13^b^	0.035
**Liver (mg/g)**					
Triacylglycerols	12.04 ± 2.48^bc^	10.14 ± 1.00^c^	28.28 ± 4.97^a^	16.62 ± 4.15^b^	< 0.001
Total cholesterol	2.97 ± 0.49^b^	2.64 ± 0.35^b^	4.19 ± 0.66^a^	2.88 ± 0.17^b^	< 0.001

Results are expressed as mean ± SD (n=5). After a one-way ANOVA, Student–Newman–Keuls (SNK) multiple comparison test, results are arranged in increasing order from left to right: a > b > c (*p* < 0.05). C, control; COA, control + *O. aurita*; HF, high fat; HFOA, high fat + *O. aurita*.

### Effect of diet treatments on plasma total lipid fatty acid composition

The plasma total lipid fatty acid composition in each group of rats is reported in Table 5. After 7 weeks of treatment, the data revealed a significant change in the plasma total lipid fatty acid composition with the different dietary treatments. *O. aurita* intake in the COA and HFOA-fed rats significantly (*p* = 0.024) increased their total *n*-3 level compared to the C and HF groups, respectively. The increased *n*-3 level was mainly related to the EPA (20:5 n-3) rate, the main polyunsaturated fatty acid (PUFA) in microalgae. Furthermore, the data revealed that the DHA (22:6 n-3) level was significantly greater in the COA and HFOA groups than in the C and HF groups respectively (*p* = 0.042). Total *n*-3 increase with *O. aurita* intake induced a decrease of the *n*-6/*n*-3 PUFA ratio in the plasma in HFOA-fed rats compared to the HF group.

**Table 5:** Plasma total lipid fatty acid composition of rats after 7 weeks of treatment

**Diet groups**	**C**	**COA**	**HF**	**HFOA**	***p *value**
**Fatty acids(% molar)**					
14:0	0.32 ± 0.07^ab^	0.34 ± 0.10^a^	0.20 ± 0.03^b^	0.23 ± 0.06^b^	0.025
16:0	17.84 ± 1.86^a^	16.94 ± 1.65^ab^	15.11 ± 0.72^bc^	14.83 ± 1.46^c^	0.010
16:1	1.51 ± 0.27^a^	1.69 ± 0.28^a^	0.56 ± 0.05^b^	0.58 ± 0.12^b^	< 0.001
18:0	9.41 ± 0.74^b^	8.76 ± 0.60^b^	12.08 ± 0.85^a^	11.29 ± 0.69^a^	< 0.001
18:1n-7 + n-9	18.60 ± 3.84	16.84 ± 2.85	15.87 ± 1.53	16.09 ± 2.39	N.S
18:2n-6	10.40 ± 0.87	10.99 ± 1.40	12.09 ± 0.96	10.98 ± 1.00	N.S
18:3n-3	0.18 ± 0.05	0.25 ± 0.04	0.24 ± 0.05	0.22 ± 0.04	N.S
20:1n-9	0.27 ± 0.06^a^	0.34 ± 0.09^a^	0.15 ± 0.03^b^	0.16 ± 0.04^b^	< 0.001
20:4n-6	23.25 ± 3.22	23.44 ± 2.96	26.64 ± 1.76	23.40 ± 3.48	N.S
20:5n-3	0.27 ± 0.07^b^	0.60 ± 0.16^a^	0.19 ± 0.05^b^	0.33 ± 0.09^b^	< 0.001
22:5n-3	0.48 ± 0.0.22	0.46 ± 0.06	0.33 ± 0.04	0.43 ± 0.07	N.S
22:6n-3	4.42 ± 1.19^ab^	5.31 ± 0.66^a^	3.64 ± 0.70^b^	4.92 ± 0.70^a^	0.042
SFA	27.58 ± 1.49	26.10 ± 1.83	27.40 ± 0.86	26.35 ± 1.77	N.S
MUFA	20.38 ± 4.14	18.87 ± 2.89	16.57 ± 1.60	16.83 ± 2.51	N.S
Total n-3	5.22 ± 1.1^ab^	5.99 ± 1.04^a^	4.22 ± 0.68^b^	5.71 ± 0.67^a^	0.024
Total n-6	33.64 ± 2.54^b^	34.44 ± 2.62^b^	38.74 ± 0.95^a^	34.39 ± 3.88^b^	0.035
MUFA / SFA	0.73 ± 0.11	0.72 ± 0.10	0.60 ± 0.07	0.64 ± 0.07	N.S
n-6 / n-3	6.63 ± 1.18^b^	5.88 ± 1.09^b^	9.35 ± 1.37^a^	6.12 ± 1.19^b^	< 0.001

Results are expressed as mean ± SD (n=5). After a one-way ANOVA, Student–Newman–Keuls (SNK) multiple comparison test, results are arranged in increasing order from left to right: a > b > c (*p* < 0.05). C, control; COA, control + *O. aurita*; HF, high fat; HFOA, high fat + *O. aurita*. N.S: Not significant.

### Effect of diet treatments on platelet aggregation, TxB_2_ production, and platelet phospholipid composition

**Figure 1:**
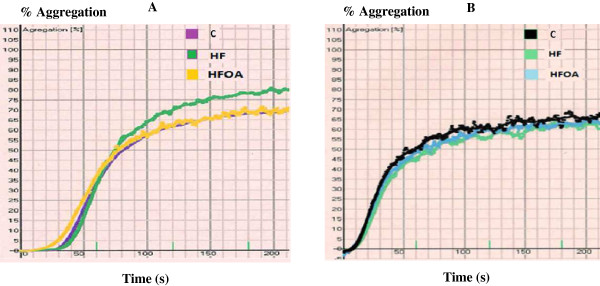
**Platelet aggregation measurement after 7 weeks of treatment with different diets.** Washed platelets were stimulated with collagen (5 μg/mL): **A** or with ADP (10 μM): **B**. C, control; HF, high fat; HFOA, high fat + *O. aurita.*

The effect of dietary treatment on ADP and collagen-induced platelet aggregation is reported in Figure 1. The maximum aggregation percentage is presented in Table 6. The data did not reveal any difference in ADP-induced platelet aggregation in the different groups. However, *O. aurita* intake in HFOA-fed rats tended to normalize the platelet aggregation induced by collagen compared to the HF-fed rats (*p* = 0.05), but this effect failed to reach statistical significance.

**Table 6:** Effect of dietary regimens on rat platelet aggregation after 7 weeks of treatment

	**% maximum aggregation**			
	**C**	**HF**	**HFOA**	***p *value**
Collagen 5 μg/mL	68.35 ± 3.56^b^	80.29 ± 3.59^a^	72.42 ± 1.09^ab^	0.053
ADP 10 μM	80.18 ± 5.72	87.63 ± 5.09	86.97 ± 4.44	N.S

Values are means ± SD of 4 independent determinations (animals). After a one-way ANOVA, Student–Newman–Keuls (SNK) multiple comparison test results are arranged in increasing order from left to right: a > b > c (*p* < 0.05). C, control; HF, high fat; HFOA, high fat + *O. aurita*. N.S: Not significant.

**Figure 2:**
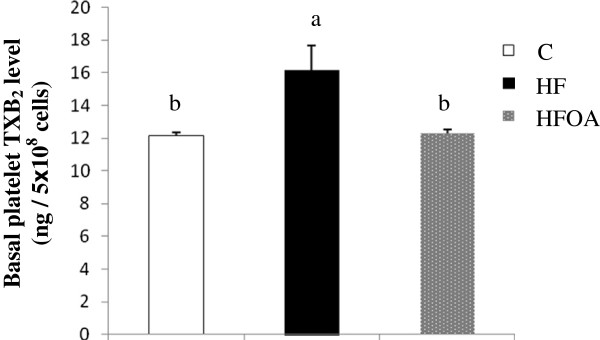
**Effect of *O. aurita *supplementation to high fat diet on the basal platelets thromboxane B2 level.** Values are expressed as the mean ± SD (n = 5). Values bearing different superscript letters are significantly different from each other. (*P* = 0.009) After a one-way ANOVA, Student–Newman–Keuls (SNK) multiple comparison test, results are arranged in increasing order from left to right: a > b > c (p < 0.05). C, control; HF, high fat; HFOA, high fat + *O. aurita.*

The effects of *O. aurita* supplementation of a high fat diet on the basal platelet level of thromboxane B2 is shown in Figure 2. The basal levels of eicosanoids in platelets are influenced by dietary fat. Rats fed with the HF diet displayed a significantly higher amount of TXB_2_ in the platelet suspension than those fed with the control diet (Figure 2). The basal TXB_2_ level in rats fed with the HFOA diet was 16.6% lower than in the HF-fed rats, which was a significant difference.

The fatty acid composition of platelet phospholipids showed that *O. aurita* intake increased the total *n*-3 PUFA level when added to the HF diet (Table 7). Increased *n*-3 in HFOA fed rats involved docosapentaenoic acid (DPA), which represents an EPA conversion product. The total *n*-6/*n*-3 ratios were also lower in the HFOA group than in HF-fed rats.

**Table 7:** Platelet phospholipids fatty acid composition of rats after 7 weeks of treatment

**Diet groups**	**C**	**HF**	**HFOA**	***p *value**
**Fatty acids(% molar)**				
14:0	0.44 ± 0.04	0.53 ± 0.10	0.59 ± 0.06	N.S
16:0	24.54 ± 0.55	24.93 ± 0.28	25.72 ± 0.68	N.S
16:1	0.50 ± 0.05^a^	0.26 ± 0.02^b^	0.25 ± 0.00^b^	< 0.001
18:0	13.90 ± 0.29	14.49 ± 0.12	14.25 ± 0.99	N.S
18:1n-7 + n-9	8.82 ± 0.20^a^	7.71 ± 0.28^b^	7.06 ± 0.33^c^	< 0.001
18:2n-6	4.33 ± 0.04	4.66 ± 0.39	4.39 ± 0.10	N.S
18:3n-3	0.49 ± 0.02	0.57 ± 0.03	0.54 ± 0.05	N.S
20:1n-9	0.46 ± 0.01	0,57 ± 0.06	0.54 ± 0.06	N.S
20:4n-6	25.88 ± 0.33^a^	23.80 ± 0.52^b^	23.80 ± 0.65^b^	0.004
20:5n-3	0.20 ± 0.03	0.17 ± 0.03	0.22 ± 0.00	N.S
22:5n-3	0.36 ± 0.06^b^	0.39 ± 0.05^b^	0.52 ± 0.03^a^	0.018
22:6n-3	0.47 ± 0.08	0.48 ± 0.05	0.63 ± 0.14	N.S
SFA	38.89 ± 0.45^b^	39.95 ± 0.22^a^	40.57 ± 0.38^a^	0.004
MUFA	9.78 ± 0.21^a^	8.55 ± 0.25^b^	7.85 ± 0.28^c^	< 0.001
Total n-3	1.52 ± 0.19^b^	1.63 ± 0.05^b^	1.92 ± 0.12^a^	0.029
Total n-6	30.21 ± 0.36^a^	28.47 ± 0.54^b^	28.19 ± 0.71^b^	0.009
MUFA / SFA	0.25 ± 0.00^a^	0.21 ± 0.00^b^	0.19 ± 0.00^c^	< 0.001
n-6 / n-3	20.02 ± 2.75^a^	17.48 ± 0.86^b^	14.71 ± 0.71^bc^	0.025

Results are expressed as mean ± SD (n=6). After a one-way ANOVA, Student–Newman–Keuls (SNK) multiple comparison test, results are arranged in increasing order from left to right: a > b > c (*p* < 0.05). C, control; HF, high fat; HFOA, high fat + *O. aurita*. N.S: Not significant.

**Figure 3:**
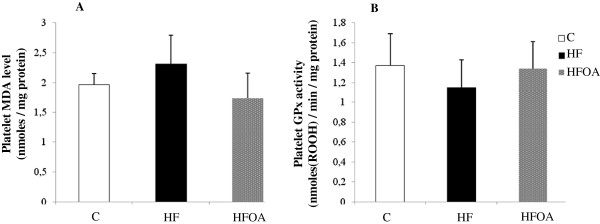
**Effect of *O. aurita *supplementation to high fat diet on platelets oxidative stress after 7 weeks of treatment.** (**A**): effect of *O. aurita* on platelets MDA level (p = 0.180); (**B**): effect of *O. aurita* on platelets GPx activity (p = 0.617). Values are expressed as the mean ± SD (n = 5). After a one-way ANOVA, Student–Newman–Keuls (SNK) multiple comparison test, results are arranged in increasing order from left to right: a > b > c (p < 0.05). C, control; HF, high fat; HFOA, high fat + *O. aurita.*

In platelets, the redox status is a critical modulator of platelet function. Figure 3 presents the MDA level and GPx activity in platelets. The data show that HF feeding tended to increase the level of MDA and decrease GPx activity in rat platelets. *O. aurita* showed a tendency to enhance platelet redox status by reducing the level of MDA and increasing GPx activity, but this effect failed to reach statistical significance.

### Effect of diet treatments on liver phospholipids and neutral lipid fraction compositions

The fatty acid compositions of the liver phospholipids (PL) and neutral lipids (NL) are reported in Tables 8 and 9 respectively. After a 7-week period on the experimental diets, the data showed that after *O. aurita* intake by COA and HFOA-fed rats, EPA incorporation into the liver phospholipid fraction was significantly greater than in the C and HF-fed rats, respectively. Thus, DPA and DHA levels in liver PL, were significantly higher in the HFOA group than in the HF-fed rats, but there was no significant difference between these PUFA rates in the COA and C groups. Furthermore, the *n*-6/*n*-3 PUFA ratio was significantly lower in the liver PL of HFOA-fed rats than in that of the HF group animals.

**Table 8:** Phospholipid fatty acid composition in liver rat after 7 weeks of diet

**Diet groups**	**C**	**COA**	**HF**	**HFOA**	***p *value**
**Fatty acids(% molar)**					
14:0	0.31 ± 0.18	0.18 ± 0.04	0.14 ± 0.03	0.16 ± 0.07	N.S
16:0	17.86 ± 1.69^a^	17.15 ± 0.90^a^	14.97 ± 0.54^b^	14.99 ± 1.14^b^	< 0.001
16:1	0.20 ± 0.07	0.16 ± 0.06	0.15 ± 0.05	0.14 ± 0.03	N.S
18:0	21.82 ± 2.05^b^	21.99 ± 1.11^b^	26.98 ± 0.39^a^	26.57 ± 0.69^a^	< 0.001
18:1n-7 + n-9	9.12 ± 1.05^a^	8.76 ± 0.51^a^	7.30 ± 0.77^b^	7.15 ± 0.68^b^	0.001
18:2n-6	8.80 ± 0.93	9.48 ± 0.96	9.65 ± 0.61	9.43 ± 0.54	N.S
18:3n-3	0.10 ± 0.03	0.10 ± 0.01	0.07 ± 0.01	0.09 ± 0.03	N.S
20:1n-9	0.14 ± 0.05	0.13 ± 0.03	0,12 ± 0.04	0.10 ± 0.03	N.S
20:4n-6	27.80 ± 1.83^ab^	26.31 ± 1.64^b^	29.37 ± 0.60^a^	28.57 ± 1.62^a^	0.044
20:5n-3	0.14 ± 0.04^bc^	0.28 ± 0.07^a^	0.12 ± 0.03^c^	0.20 ± 0.03^b^	< 0.001
22:5n-3	0.57 ± 0.08^b^	0.66 ± 0.09^ab^	0.61 ± 0.06^b^	0.71 ± 0.09^a^	0.038
22:6n-3	7.79 ± 0.50^a^	7.80 ± 0.30^a^	6.94 ± 0.60^b^	7.46 ± 0.39^ab^	0.025
SFA	39.99 ± 0.83^b^	39.47 ± 0.59^b^	42.14 ± 0.67^a^	41.77 ± 0.67^a^	< 0.001
MUFA	9.65 ± 1.00^a^	9.04 ± 0.55^a^	6.83 ± 0.90^b^	7.62 ± 0.75^b^	< 0.001
Total n-3	8.47 ± 0.49^a^	8.81 ± 0.47^a^	7.68 ± 0.65^b^	8.35 ± 0.29^a^	0.009
Total n-6	36.60 ± 1.03^b^	37.57 ± 0.95^ab^	38.99 ± 0.5^a^	38.00 ± 1.34^ab^	0.025
Δ9 desaturase index	0.29 ± 0.04^a^	0.29 ± 0.02^a^	0.20 ± 0.03^b^	0.21 ± 0.02^b^	0.002
MUFA / SFA	0.25 ± 0.04^a^	0.23 ± 0.02^a^	0.17 ± 0.02^b^	0.17 ± 0.01^b^	< 0.001
n-6 / n-3	4.32 ± 0.18^b^	4.15 ± 0.30^b^	5.13 ± 0.52^a^	4.55 ± 0.24^b^	0.002

Results are expressed as mean ± SD (n=5). After a one-way ANOVA, Student–Newman–Keuls (SNK) multiple comparison test, results are arranged in increasing order from left to right: a > b > c (*p* < 0.05). C, control; COA, control + *O. aurita*; HF, high fat; HFOA, high fat + *O. aurita*. N.S: Not significant. Δ9 desaturase index = [18:1n-7 + n-9/(18:0 + 18:1n-7 + n-9)].

**Table 9:** Liver neutral lipids fatty acid composition of rats after 7 weeks of treatment

**Diet groups**	**C**	**COA**	**HF**	**HFOA**	***p *value**
**Fatty acids(% molar)**					
14:0	0.95 ± 0.06^a^	0.88 ± 0.32^a^	0.51 ± 0.17^b^	0.64 ± 0.29^ab^	0.030
16:0	25.53 ± 2.22	24.39 ± 2.73	22.84 ± 0.55	23.07 ± 1.80	N.S
16:1	3.75 ± 1.00^a^	3.41 ± 0.74^a^	0.34 ± 0.03^b^	0.40 ± 0.07^b^	< 0.001
18:0	6.06 ± 1.76	5.75 ± 2.58	5.42 ± 1.32	4.39 ± 0.86	N.S
18:1n-7 + n-9	35.06 ± 4.60^b^	35.84 ± 3.57^b^	32.97 ± 1.90^b^	41.00 ± 2.48^a^	0.005
18:2n-6	11.41 ± 1.96^b^	11.20 ± 2.91^b^	15.14 ± 1.57^a^	14.61 ± 1.36^a^	0.008
18:3n-3	0.46 ± 0.20	0.54 ± 0.16	0.62 ± 0.06	0.65 ± 0.10	N.S
20:1n-9	0.14 ± 0.01^c^	0.40 ± 0.06^a^	0.15 ± 0.00^c^	0.28 ± 0.00^b^	< 0.001
20:4n-6	3.89 ± 0.45	3.39 ± 0.81	3.27 ± 0.78	3.64 ± 1.44	N.S
20:5n-3	0.13 ± 0.04^b^	0.33 ± 0.16^a^	0.15 ± 0.04^b^	0.19 ± 0.05^b^	0.011
22:5n-3	0.16 ± 0.03^b^	0.35 ± 0.10^a^	0.12 ± 0.01^b^	0.41 ± 0.15^a^	< 0.001
22:6n-3	1.09 ± 0.07^b^	1.58 ± 0.17^a^	1.02 ± 0.19^b^	1.14 ± 0.06^b^	0.004
SFA	32.75 ± 1.26^a^	31.02 ± 2.41^a^	28.77 ± 1.65^b^	28.72 ± 0.9^b^	0.002
MUFA	38.88 ± 4.19^ab^	42.15 ± 5.39^a^	33.60 ± 2.06^b^	42.97 ± 4.00^a^	0.007
Total n-3	1.88 ± 0.16^b^	2.68 ± 0.43^a^	1.94 ± 0.27^b^	2.78 ± 0.68^a^	0.043
Total n-6	14.96 ± 2.01	14.14 ± 2.48	20.40 ± 4.18	19.52 ± 3.55	N.S
Δ9 desaturase index	0.85 ± 0.05	0.86 ± 0.05	0.86 ± 0.03	0.89 ± 0.03	N.S
MUFA / SFA	1.19 ± 0.15^b^	1.55 ± 0.24^a^	1.17 ± 0.06^b^	1.49 ± 0.09^a^	0.004
n-6 / n-3	7.37 ± 0.94^ab^	5.56 ± 0.60^c^	8.44 ± 0.84^a^	7.25 ± 0.66^b^	0.001

Results are expressed as mean ± SD (n=5). After a one-way ANOVA, Student–Newman–Keuls (SNK) multiple comparison test, results are arranged in increasing order from left to right: a > b > c (*p* < 0.05). C, control; COA, control + *O. aurita*; HF, high fat; HFOA, high fat + *O. aurita*. N.S: Not significant. Δ9 desaturase index = [18:1n-7 + n-9/(18:0 + 18:1n-7 + n-9)].

Compared to the liver PL fraction, more modifications were revealed in the composition of NL (Table 9). Beside total *n*-3 enrichment in the liver NL fraction, especially with EPA, DPA, and DHA, the data showed that the total monounsaturated fatty acid (MUFA) level, especially of 16:1n-7 and 18:1n7 + 18:1n-9 MUFA levels, was significantly higher in the COA and HFOA groups than in the C and HF groups. The *n*-6/*n*-3 PUFA ratio was also lower in the liver NL fraction in COA and HFOA-fed rats than in the C and HF groups.

### Effect of diet treatments on the level of MDA and on GPx activity in liver

**Figure 4:**
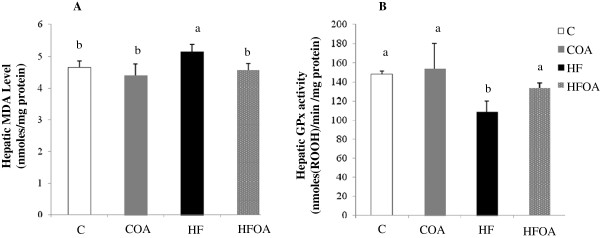
**Effect of *O. aurita *supplementation of standard or high fat diets on hepatic oxidative stress after 7 weeks of treatment.** (**A**): effect of *O. aurita* on hepatic MDA level (p = 0.006); (**B**): effect of *O. aurita* on hepatic GPx activity (*p* = 0.002). Values are expressed as the mean ± SD (n = 5). Values bearing different superscript letters are significantly different from each other. After a one-way ANOVA, Student–Newman–Keuls (SNK) multiple comparison test, the results are arranged in increasing order from left to right: a > b > c (p < 0.05). C, control; COA, control + *O. aurita*; HF, high fat; HFOA, high fat + *O. aurita.*

The hepatic MDA level and GPx activity are shown in Figure 4. A significantly higher liver MDA level was observed in the HF group than in the C group. *O. aurita* intake in HFOA-fed rats resulted in a significantly lower MDA level in the liver than in HF-fed rats. Thus, after 7 weeks of treatment, the HF diet had induced a significantly lower level of hepatic GPx activity than the C diet. However, HF diet supplementation with *O. aurita* resulted in significantly greater antioxidant activity of GPx in the liver than that observed with the HF diet.

## Discussion

The present investigation was undertaken to assess the influence of *O. aurita*, a marine diatom rich in EPA, on the risk factors for high-fat induced MS in rats. In this study, the HF diet group rats had significantly higher final body weight, adiposity, blood glucose, TG and total cholesterol levels than were found in the animals in the C group. Abdominal obesity and insulin resistance have been proposed as being the main causal factors of MS [38]. It was also found that *O. aurita* intake in HFOA group decreased adipose tissue weight, plasma glucose, and TG and total cholesterol levels, but had no effect on final body weight in the COA or HFOA groups as compared to the C and HF groups respectively. These results suggest that the administration of *O. aurita* reduces MS risk factors by lowering glycemia and blood lipid levels. Marine organisms such as fish and microalgae are known to be rich in *n*-3 fatty acids, and specifically in EPA and DHA. Previous studies have already reported that purified EPA or DHA have differing effects on parameters involved in cardiovascular disease prevention [15]. Matzusawa et al. [38] showed that *n*-3 PUFA plays an important role in preventing obesity by acting on the adipocytokines that regulates lipid and glucose homeostasis. Itoh et al. [39] reported that dietary EPA increased adiponectin secretion in genetically and high-fat diet induced obese mice. Indeed, the consumption of fish oil enriched in EPA can markedly reduce the levels of plasma triacylglycerols and associated very-low-density lipoprotein (VLDL) in the circulation in healthy subjects and selected patients with hypertriglyceridemia [40]. Nemoto et al. [18] showed that EPA markedly reduced the fatty droplets in the liver cells, and also lowered plasma levels of total cholesterol and triacylglycerol in high fat-fed mice.

Platelet aggregation is considered to be closely involved in the development of arterial thrombosis after the formation of atherosclerotic plaques [41]. Furthermore, a variety of platelet functions abnormalities, including increased adhesiveness and activation *in vitro* and *in vivo* and increased sensitivity to physiological agonists, have been identified in obese subjects [42]. The polyunsaturated fatty acids EPA and DHA play a crucial role in reducing platelet aggregation [43]. In the present investigation we compared platelet activities in three groups: control, C, high-fat, HF, and high fat + *O. aurita,* HFOA. It was found that rats fed the HFOA diet displayed lower collagen-induced platelet aggregation, but not in ADP-induced platelet aggregation, than rats fed with the HF diet. The decrease in platelet aggregation in the HFOA group was confirmed by the lower TXB_2_ level in this group compared to the HF group. The fatty acid composition of platelet phospholipids revealed higher levels of *n*-3 PUFA in the HFOA than in the HF group. These findings could be explained by the incorporation and conversion of EPA produced by the microalgae in the platelet phospholipids. When *n*-3 PUFA are consumed they are selectively incorporated into the phospholipids of the platelet membrane, and thus decrease the release and formation of TXB_2_, which is a potent inducer of platelet aggregation. Burri et al. [44] have shown that the consumption of a diet high in linoleic acid (LA) decreases ADP- and collagen-induced platelet aggregation compared to a diet containing oleic acid. Studies by Kwon et al. [45] have found that the consumption of both oleic acid and linoleic acid reduce collagen-induced platelet aggregation compared to that found in subjects consuming a diet high in saturated fatty acids. Moreover, Véricel et al. [46] showed that a small intake of *n*-3 PUFA reduced the oxidative stress in the platelets of elderly people, and could be beneficial to subjects with atherothrombotic tendencies by lowering the cell peroxide tone.

The levels of *n*-3 fatty acids incorporated into tissue lipids can be considered to be a cardiovascular disease risk marker, as has often been reported previously [47,48]. The results reported in Tables 5, 7, 8 and 9 showed higher levels of *n-*3 PUFA in the plasma and liver fatty acid compositions in rats fed with COA and HFOA diets than those in rats on the fed with C or HF diets. These results show that the EPA provided by the *O. aurita* diet is incorporated into the plasma and liver lipids. Moreover, there is a relationship between food intake and lipid enrichment with EPA. The rats fed the COA diet had a higher food intake than those receiving the HFOA diet, and higher EPA levels were observed in the plasma and liver in the COA group than in the HFOA group.

The liver is a very important site for maintaining body lipid homeostasis. Our data show that rats fed with the HF diet had higher hepatic TG and total cholesterol levels than HFOA-fed rats, which suggests that *O. aurita* could regulate hepatic genes involved in lipid metabolism through the effect of EPA. Stearoyl-CoA desaturase (SCD) is an endoplasmic reticulum enzyme that catalyzes the biosynthesis of monounsaturated fatty acids (MUFAs) from saturated fatty acids (SFAs) that are either synthesized *de novo* or derived from the diet. The regulation of this process plays a critical role in disorders such as obesity, diabetes, and atherosclerosis. SCD1, the main SCD isoform expressed in liver, is a key player in the regulation of lipid partitioning in liver. The importance of SCD1 in neutral lipid synthesis in liver has been confirmed by studies in SCD1 -/- mice, in which an SCD1 deficiency leads to a fall in the hepatic triacylglycerol and cholesterol ester contents [49,50] and down regulates *de novo* fatty acid synthesis [51]. The results shown in Table 9 show a lower level of total MUFA in liver neutral lipids in the HF group than in the controls. However, the lower MUFA level was enhanced by *O. aurita* intake in HFOA fed rats. These results may suggest the potential regulation of SCD1 by *O. aurita*. Velliquette et al. [52] showed that diets enriched in linoleic acid, α-linolenic acid and, by metabolic inference, EPA, can regulate SCD activity at the level of transcription.

Numerous studies have shown that oxidative stress is increased in obesity, which is an essential component of MS, and that reactive oxygen species (ROS) levels can be reduced as a result of weight loss [53]. Obesity induced by a high fat diet leads to enhanced oxidative stress in rats [54]. Roberts et al. [55] also showed that ROS were overproduced in a rat model of MS induced by a diet high in fat and refined sugar. The results shown in Figure 3 showed that *O. aurita* intake in HFOA group resulted in significantly lower hepatic oxidative stress after 7 weeks of treatment than in HF fed rats. Rats fed with HFOA had lower level of hepatic MDA than HF fed rats. Thus, the antioxidant activity of hepatic GPx was higher in the HFOA group than in the HF group. Indeed, a significant correlation (r = 0.986 and *p* = 0.002) has been established between a fall in MDA level and an increase in GPx activity in response to the HFOA diet. These results prove that *O. aurita* supplementation of the HF diet reduces the hepatic oxidative stress after 7 weeks of treatments. Beside *n*-3 PUFA, *O. aurita* also contains a large portion of pigments. Chlorophyll *a* and fucoxanthin are the major pigments in *O. aurita* (30.0 and 20.4 % of total pigments, respectively). Many studies have reported that micronutrients, such as polyphenols, pigments, vitamins and minerals, can prevent or at least attenuate the damage caused by oxidative stress. The Feillet-Coudray et al. [56] study demonstrates that a polyphenol extract seems to have some protective effects against hepatic steatosis and against the oxidative stress induced by high fat, high sucrose diet in rats. Fucoxanthin, like other carotenoids displays remarkable bioactivities (antioxidant, anti-obesity etc.) that are relevant to human health (for a review see Peng et al. [57]).

The present investigation showed that *O. aurita* could be used as food supplement to prevent risk factors for high-fat induced MS. The beneficial effects of *O. aurita* showed in the present investigation could be due to a synergistic effect between the various different active microalgal components, such as *n*-3 PUFA and pigments. Kay [58] proposed that the possible active ingredients of *Chlorella* that reduce blood lipid levels could be water-soluble fiber, vegetable protein, phospholipids, Vitamin C, Vitamin E and beta-carotene. Furthermore numerous authors have provided evidence that a combination of phytosterols and *n*-3 fatty acids may reduce cardiovascular risk in a complementary and synergistic way [59,60].

In conclusion, our data show that *O. aurita* supplementation reduces the risk factors for high-fat induced MS in rats after 7 weeks of treatment. *O. aurita* intake in HFOA-fed rats resulted in lower levels of glycemia, triacylglycerols, and total cholesterol in plasma and liver than the same levels found in HF-fed rats. Furthermore, rats given the HFOA diet tended to have lower collagen-induced platelet aggregation, decreased platelet TXB_2_ level and enhanced *n*-3 PUFA incorporation in plasma, platelet, and liver lipids. Finally, *O. aurita* as food supplement in HFOA-fed rats displayed an antioxidant effect in the liver, with lower levels of MDA and increased GPx activity.

## Abbreviations

MS: Metabolic syndrome; CVD: Cardiovascular diseases; PUFA: Polyunsaturated fatty acid; *n*-3: Omega-3; *n*-6: Omega-6; EPA: Eicosapentaenoic acid; DHA: Docosahexaenoic acid; PPARs: Peroxisome proliferator-activated receptors; EPA-E: Eicosapentaenoic acid ethyl ester; SREBP-1: Sterol regulatory element binding protein-1; SCD1: Stearoyl-CoA desaturase 1; PRP: Platelet rich plasma; PPP: Platelet poor plasma; PL: Phospholipids; NL: Neutral lipids; FAMEs: Fatty acid methyl ester; MDA: Malondialdehyde; GPx: Glutathione peroxidase; TXB_2_: Thromboxane B_2_; AT: Adipose tissue; Bw: Bodyweight; TG: Triacylglycerol; VLDL: Very-low-density lipoprotein; LA: Linoleic acid; MUFA: Monounsaturated fatty acids; SFA: Saturated fatty acids; ROS: Reactive oxygen species.

## Competing interests

The authors declare that they have no competing interests.

## Authors’ contributions

AH participated in planning the study, the experimental work, analysis of samples, statistical analysis, data analysis and manuscript preparation. LU participated in planning the study, the experimental work, statistical analysis and manuscript preparation. VM participated in planning the study, the experimental work and manuscript preparation. FG participated in the experimental work. FPV participated in aggregation experiments, result discussion and analysis and in manuscript preparation. NM participated in the planning and organization of the study, in the experimental work and manuscript preparation. GT participated in the planning and organization of the study and manuscript preparation. All the authors have read and approved the final manuscript.

## References

[B1] Fulop T Tessier D Carpentier A The metabolic syndrome Pathol Biol 2006 54 375 386 10.1016/j.patbio.2006.07.002 16904849

[B2] Cheng JY Shih MF Preventing dyslipidemia by Chlorella pyrenoidosa in rats and hamsters after chronic high fat diet treatment Life Sci 2005 76 3001 3013 10.1016/j.lfs.2004.10.055 15850594

[B3] Li W Shi YH Yang RL Cui J Xiao Y Wang B Le Wei G Effect of somatostatin analog on high-fat diet-induced metabolic syndrome: involvement of reactive oxygen species Peptides 2010 31 625 629 10.1016/j.peptides.2009.11.008 19931331

[B4] Samane S Christon R Dombrowski L Turcotteb S Charrouf Z Lavigne C Levy E Bachelar H Amarouch H Marette A Haddad PS Fish oil and argan oil intake differently modulate insulin resistance and glucose intolerance in a rat model of dietary-induced obesity Metab Clin Exp 2009 58 909 919 10.1016/j.metabol.2009.02.013 19394055

[B5] Kajikawa S Harada T Kawashima A Imada K Mizuguchi K Highly purified eicosapentaenoic acid prevents the progression of hepatic steatosis by repressing monounsaturated fatty acid synthesis in high-fat/high-sucrose diet-fed mice Prostagland Leukotr Essent Fatty Acids 2009 80 229 238 10.1016/j.plefa.2009.02.004 19328666

[B6] Pérez-Echarri N Pérez-Matute P Marcos-Gómez B Marti A Martinez JA Moreno-Aliaga MJ Down-regulation in muscle and liver lipogenic genes: EPA ethyl ester treatment in lean and overweight (high-fat-fed) rats J Nutr Biochem 2009 20 705 714 10.1016/j.jnutbio.2008.06.013 18829285

[B7] Adan Y Shibata K Sato M Ikeda I Imaizumi K Effects of Docosahexaenoic and Eicosapentaenoic acid on lipid metabolism, eicosanoid production, platelet aggregation and atherosclerosis in hypercholesterolemic rats Biosci Biotechnol Biochem 1999 63 11 119 10.1271/bbb.63.111 10052130

[B8] Hee Hong J Seon Lee I Effects of Artemisia capillaris ethyl acetate fraction on oxidative stress and antioxidant enzyme in high-fat diet induced obese mice Chem Biol Interact 2009 179 88 93 10.1016/j.cbi.2008.12.002 19121296

[B9] Makni M Fetoui H Garoui EM Gargoui NK Jaber H Makni J Boudawara T Zeghal N Hypolipidemic and hypatoprotective seeds mixture diet rich in ω-3 and ω-6 fatty acids Food Chem Toxicol 2010 48 2239 2246 10.1016/j.fct.2010.05.055 20510326

[B10] Cameron AJ Shaw JE Zimmet PZ The metabolic syndrome: prevalence in worldwide populations Endocrinol Metab Clin North Am 2004 33 351 375 10.1016/j.ecl.2004.03.005 15158523

[B11] Minich DM Bland JS Dietary management of the metabolic syndrome beyond macronutriments Nutr Rev 2008 66 429 444 10.1111/j.1753-4887.2008.00075.x 18667004

[B12] Lyer A Panchal S Poudyal H Brown L Potential health benefits of Indian spices in the symptoms of the metabolic syndrome: a review Indian J Biochem Biophys 2009 46 467 481 20361710

[B13] Paniagua JA Pérez-Martinez P Gjelstad IMF Tierney AC Delgado-Lista J Defoort C Blaak EE Risérus ULF Drevon CA Kiec-Wilk B Lovegrove JA Roche HM López-Miranda J LIPGENE Study Investigators A low-fat high-carbohydrate diet supplemented with long-chain n-3 PUFA reduces the risk of the metabolic syndrome Atherosclerosis 2011 218 443 450 10.1016/j.atherosclerosis.2011.07.003 21839455

[B14] Balk EM Lichtenstein AH Chung M Kupelnick B Chew P Lau J Effects of omega-3 fatty acids on serum markers of cardiovascular diseases risk: a systematic review Atherosclerosis 2006 189 19 30 10.1016/j.atherosclerosis.2006.02.012 16530201

[B15] Mozaffarian D Wu JHY Omega-3 fatty acids and cardiovascular disease J Am Coll Cardiol 2011 58 2047 2067 10.1016/j.jacc.2011.06.063 22051327

[B16] Lerman RH Kaskel L McIntosh M Najm W Fernandez ML Baruffi E Harris E Correction of the omega-3 index in women with metabolic syndrome by adding omega-3 supplements to a mediterranean style diet J Clin Lipidol 2011 5 s146

[B17] Adkins Y Kelley DS Mechanisms underlying the cardioprotective effects of omega-3 polyunsaturated fatty acids J Nutr Biochem 2010 21 781 792 10.1016/j.jnutbio.2009.12.004 20382009

[B18] Nemoto N Suzuki S Kikuchi H Okabe H Sassa S Sakamoto S Ethyl-eicosapentaenoic acid reduces liver lipids and lowers plasma levels of lipids in mice fed a high-fat diet In Vivo 2009 23 685 690 19779101

[B19] Poudyal H Panchal SK Diwan V Brown L Omega-3 fatty acids and metabolic syndrome: effects and emerging mechanisms of action Prog Lipid Res 2011 50 372 387 10.1016/j.plipres.2011.06.003 21762726

[B20] Judé S Roger S Martel E Besson P Richard S Bougnoux P Champeroux P Le Guennec JY Dietary long-chain omega-3 fatty acids of marine origin: a comparison of their protective effects on coronary heart diseases and breast cancers Prog Biophys Mol Biol 2006 90 299 325 10.1016/j.pbiomolbio.2005.05.006 16005051

[B21] Belarbi H Molina E Chisti YA Process for high yield and scaleable recovery of high purity eicosapentaenoic acid esters from microalgae and fish oil Proc Biochem 2000 35 951 969 10.1016/S0032-9592(00)00126-6 10771055

[B22] Borowitzka MA Micro-algae as sources of fine chemicals Microbiol Sci 1986 3 372 375 3153571

[B23] Shibata S Natori Y Nishihara T Tomisaka K Matsumoto K Sansawa H Nguyen VC Antioxydant and anticataract effects of Chlorella on rats with streptozotocin-induced diabetes J Nutr Sci Vitaminol 2003 49 334 339 10.3177/jnsv.49.334 14703308

[B24] Sano T Tanaka Y Effects of dried powdered Chlorella vulgaris on experimental atherosclerosis and alimentary hypercholesterolemia in cholesterol-fed rabbit Artery 1987 14 76 84 3566534

[B25] Lee SH Kang HJ Lee HJ Kang MH Park YK Six-week supplementation with Chlorella has favorable impact on antioxidant status in Korean male smokers Nutrition 2010 26 175 183 10.1016/j.nut.2009.03.010 19660910

[B26] Okudo M Hasegawa T Sonoda M Okabe T Tanaka M The effects of Chlorella on the level of cholesterol in serum and liver Jpn J Nutr 1975 33 3 8 10.5264/eiyogakuzashi.33.3

[B27] Moreau D Tomasoni C Cathrine J Kaas R Le Guedes R Cadoret JP Muller-Feuga A Kontiza I Vagias C Roussis V Roussakis C Cultivated microalgae and the carotenoid fucoxanthin from Odontella aurita as potent anti-proliferative agents in bronchopulmonary and epithelial cell lines Envir Toxicol Pharmacol 2006 22 97 103 10.1016/j.etap.2006.01.004 21783694

[B28] Lattimer JM Haub MD Effects of dietary fibers and its components on metabolic health Nutrients 2010 2 1266 1289 10.3390/nu2121266 22254008 PMC3257631

[B29] Mekhfi H El Haouari M Legssyer A Bnouham M Aziz M Atmani F Remmal A Ziyyat A Platelet anti-aggregant property of some Moroccan medicinal plants J Ethnopharmacol 2004 94 317 322 10.1016/j.jep.2004.06.005 15325737

[B30] Born GVR Aggregation of blood platelets by adenosine diphosphate and its reversal Nature 1962 194 927 929 13871375 10.1038/194927b0

[B31] Folch J Lees M Sloane Stanley GH A simple method for the isolation and purification of total lipids from animal tissues J Biol Chem 1957 226 497 509 13428781

[B32] Bligh EG Dyer WJ A rapid method of lipid extraction and purification Can J Biochem Physiol 1959 37 911 917 10.1139/o59-099 13671378

[B33] Slover HT Lanza E Quantitative analysis of food fatty acids by capillary gas chromatography J Am Oil Chem Soc 1979 56 933 943 10.1007/BF02674138

[B34] Ohkawa H Ohishi N Yagik K Assay for lipid peroxides in animal tissues by thiobarbituric acid reaction Anal Biochem 1979 95 351 358 10.1016/0003-2697(79)90738-3 36810

[B35] Paglia DE Valentine WN Studies on the quantitative and qualitative characterization of erythrocyte glutathione peroxidase J Lab Clin Med 1967 70 158 169 6066618

[B36] Chaudiére J Gerard D Douste-Blazy L, Mendy F Dosage de l’activité glutathion-peroxydase Biologie des Lipides chez l'Homme 1988 Paris: Médicales Internationales 275 289

[B37] Bradford MM A rapid and sensitive method for the quantitation of microgram quantities of protein utilizing the principle of protein dye-binding method Anal Biochem 1976 72 248 254 10.1016/0003-2697(76)90527-3 942051

[B38] Matsuzawa Y Fumahashi T Nakamura T Molecular mechanism of metabolic syndrome X, contribution of adipocytokines: adipocyte-derived bioactive substance Ann NY Acad Sci 1999 892 146 154 10.1111/j.1749-6632.1999.tb07793.x 10842660

[B39] Itoh M Suganami T Satoh N Tanimoto-Koyama K Yuan X Tanaka M Kawano H Yano T Aoe S Takeya M Shimatsu A Kuzuya H Kamei Y Ogawa Y Increased adiponectin secretion by highly purified eicosapentaenoic acid in rodent models of obesity and human obese subjects Arterioscler Thromb Vasc Biol 2007 27 1918 1925 10.1161/ATVBAHA.106.136853 17569885

[B40] Phillipson BE Rothrock DW Connor WE Harris WS Illingworth DR Reduction of plasma lipids, lipoproteins, and apoproteins by dietary fish oils in patients with hypertriglyceridemia N Engl J Med 1985 312 1210 1216 10.1056/NEJM198505093121902 3990714

[B41] Fuster V Chesebro JH Antithrombotic therapy: role of platelet-inhibitor drugs. II. Pharmacologic effects of platelet-inhibitor drugs (second of three parts) Mayo Clin Proc 1981 56 s185 7010003

[B42] Anfossi G Russo I Trovati M Platelet dysfunction in central obesity Nutr Metab Cardiovasc Dis 2009 19 440 449 10.1016/j.numecd.2009.01.006 19346117

[B43] Lagarde M Calzada C Guichardant M Véricel E Dose effect and metabolism of docosahexaenoic acid: Pathophysiological relevance in blood platelets Prostagland Leukotr Essent Fatty Acids in press 10.1016/j.plefa.2012.04.001 22520055

[B44] Burri BJ Dougherty RM Kelley DS Iacono JM Platelet aggregation in humans is affected by replacement of dietary linoleic acid with oleic acid Am J Clin Nutr 1991 54 359 362 1858699 10.1093/ajcn/54.2.359

[B45] Know J Snook JT Wardlaw GM Hwang DH Effects of diets high in saturated fatty acids, canola oil, or safflower oil on platelet function, thromboxane B2 formation, and fatty acid composition of platelet phospholipids Am J Clin Nutr 1991 54 351 358 1677525 10.1093/ajcn/54.2.351

[B46] Véricel E Calzada C Chapuy P Lagarde M The influence of n-3 fatty acids on platelets in elderly people Atherosclerosis 1999 147 187 192 10.1016/S0021-9150(99)00171-9 10525140

[B47] Arkhipenko YV Sazontova TG Mechanisms of the cardioprotective effect of a diet enriched with n-3 polyunsaturated fatty acids Pathophysiol 1995 2 131 140 10.1016/0928-4680(95)00017-U

[B48] Zampolli A Bysted A Leth T Mortensen A De Caterina R Falk E Contrasting effect of fish oil supplementation on the development of atherosclerosis in murine models Atherosclerosis 2006 184 78 85 10.1016/j.atherosclerosis.2005.04.018 15946668

[B49] Miyazaki M Kim YC Ntambi JM A lipogenic diet in mice with a disruption of stearoyl-CoA desatuase 1 gene reveals a stringent requirement of endogenous monounsaturated fatty acids for triglyceride synthesis J Lipid Res 2001 42 1018 1024 11441127

[B50] Miyazaki M Kim YC Gray-Keller MP Attie AD Ntambi JM The biosynthesis of hepatic cholesterol esters and triglycerides is impaired in mice with a disruption of the gene for stearoyl-CoA desaturase 1 J Biol Chem 2000 275 30132 30138 10899171 10.1074/jbc.M005488200

[B51] Ntambi JM Miyazaki M Stoehr JP Lan H Kendziorski CM Yandell BS Song Y Cohen P Friedman JM Attie AD Loss of stearoyl-CoA desaturase-1 function protects mice against adiposity Proc Natl Acad Sci U S A 2002 99 11482 11486 10.1073/pnas.132384699 12177411 PMC123282

[B52] Velliquette RA Gillies PJ Kris-Etherton MP Green JW Zhao G Vanden Heuvel JP Regulation of human stearoyl-CoA desaturase by omega-3 and omega-6 fatty acids: Implications for the dietary management of elevated serum triglycerides J Clin Lipidol 2009 3 281 288 10.1016/j.jacl.2009.06.002 21291825

[B53] Vincent HK Taylor AG Biomarkers and potential mechanisms of obesity-induced oxidant stress in humans Int J Obes 2006 30 400 418 10.1038/sj.ijo.0803177 16302012

[B54] Dobrian AD Davies MJ Schriver SD Lauterio TJ Prewitt RI Oxidative stress in a rat model of obesity-induced hypertension Hypertension 2011 37 554 560 11230334 10.1161/01.hyp.37.2.554

[B55] Roberts CK Barnard RJ Sindhu RK Jurczak M Ehdaie A Vaziri ND Oxidative stress and dysregulation of NAD(P)H oxidase and antioxidant enzymes in diet-induced metabolic syndrome Metabolism 2006 55 928 934 10.1016/j.metabol.2006.02.022 16784966

[B56] Feillet-Coudray C Sutra T Fouret G Ramos J Wrutniak-Cabello C Cabello G Cristol JP Coudray C Oxidative stress in rats fed a high-fat high-sucrose diet and preventive effect of polyphenols: involvement of mitochondrial and NAD(P)H oxidase systems Free Radic Biol Medic 2009 46 624 632 10.1016/j.freeradbiomed.2008.11.020 19135522

[B57] Peng J Yuan JP Wu CF Wang JH Fucoxanthin, a marine carotenoid present in brown seaweeds and diatoms: metabolism and bioactivities relevant to human health Mar Drugs 2011 9 1806 1828 10.3390/md9101806 22072997 PMC3210606

[B58] Kay PA Microalgae as food and supplement Crit Rev Food Sci Nutr 1991 30 555 573 10.1080/10408399109527556 1741951

[B59] Micallef MA Garg ML Beyond blood lipids: phytosterols, statins and omega-3 polyunsaturated fatty acid therapy for hyperlipidemia J Nutr Biochem 2009 20 927 939 10.1016/j.jnutbio.2009.06.009 19733044

[B60] Rao AV Rao LG Carotenoids and human health Pharmacol Res 2007 55 207 216 10.1016/j.phrs.2007.01.012 17349800

